# Fulminant parvovirus B19 myocarditis after chemotherapy: full recovery after antiviral therapy with tenofovir

**DOI:** 10.1007/s00392-021-01955-3

**Published:** 2021-10-20

**Authors:** Tobias Koenig, Tibor Kempf, Heinz-Peter Schultheiss, Markus Cornberg, Johann Bauersachs, Andreas Schäfer

**Affiliations:** 1 Department of Cardiology and Angiology, Hannover Medical School, Carl-Neuberg-Str. 1, 30625 Hannover, Germany; 2 Institute for Cardiac Diagnostics and Therapy, IKDT GmbH, Berlin, Germany; 3 Department of Gastroenterology, Hepatology and Endocrinology, Hannover Medical School, Hannover, Germany

Sirs:

A 19-year-old female patient presented to the emergency department with fever, tachycardia, dyspnea, and abdominal pain. She was diagnosed with an ovarian germ cell tumor 1 month previously. Chemotherapy had been initiated including cisplatin, etoposide, and bleomycin 2 weeks before. Transthoracic echocardiography showed normal left ventricular (LV) and right ventricular function prior to chemotherapy.

On admission, physical examination and electrocardiogram did not show relevant pathologies. Heart rate was 120 beats per minute (sinus rhythm), blood pressure was 101/58 mmHg, and temperature was 38.5 °C. Laboratory analyses revealed markedly elevated C-reactive protein (302 mg/L, reference value < 6 mg/L) and procalcitonin (9.6 µg/L, reference value < 0.1 µg/L), and severe leukocytopenia (100/µL, reference value 3600 to 10,500/µL). A transthoracic echocardiogram showed severely impaired systolic LV function and LV dilatation, and preserved systolic right ventricular function (Supplemental Video 1). There was moderate to severe mitral regurgitation. Chest X-ray demonstrated pulmonary congestion and a left-sided pleural effusion.

The patient’s status rapidly deteriorated and she developed severe cardiogenic shock (serum lactate 2.5 mmol/L, LV end-diastolic pressure 21 mmHg) requiring high-dose vasopressors and inotropes. NT-pro BNP level was 29,390 ng/L, hs-Troponin T level was 66 ng/L. The patient was urgently transferred to the catheterization laboratory. Coronary angiography revealed normal coronary arteries. Endomyocardial biopsies (EMB) from the LV were obtained and an Impella CP micro-axial flow-pump (Abiomed, Danvers, Massachusetts, USA) was inserted for LV unloading [1].

The patient was intubated and mechanically ventilated due to progressive respiratory failure despite rapid stabilization of hemodynamics on mechanical support. Levosimendan was administered to gradually reduce vasopressors and inotropes. After 19 h on Impella support, inotropes were weaned and early intravenous heart failure treatment was started using the short-acting β-blocker landiolol due to its almost neutral effect on blood pressure. Antibiotic treatment was applied due to bacteremia with *Escherichia coli* and *Streptococcus salivarius*. High-dose corticosteroids were given for 3 consecutive days, but without significant clinical benefit.

Histologic, immunohistologic, molecular, and virological analyses of the EMBs (Fig. 1) were performed. Molecular diagnostics for common cardiotropic viruses were made by PCR testing [2, 3]. In addition to diagnostics of B19V genomes, B19V viral activity was assessed [4]. Two quantitative PCR (qPCR) assays specifically amplifying the B19V non-structural (NS1) and capsid protein (VP1/2) sequences were applied to establish diagnosis of active viral infection. A viral load of 1926 ± 1104 copies per µg genomic DNA was measured. Sequencing of a VP1/2 fragment could identify infection with B19V genotype 1. Detection of viral RNA as a sign of viral transcriptional activity was confirmed for both viral transcripts, the NS1 (156 ± 35 transcripts per µg total RNA) and VP1/2 sequences (831 ± 42 transcripts per µg total RNA). Co-infection with other cardiotropic viruses and systemic virus infection were excluded. For the evaluation of morphological features and detection of myocarditis, four standard stains were used (Heidenhain’s AZAN trichrome stain, haematoxylin and eosin stain, elastic Van Gieson stain, and periodic acid–Schiff stain) (Fig. 2). Immunohistochemistry was used to characterize inflammatory infiltration. Myocardial inflammation was present with 14.0 lymphocytes/mm^2^. Macrophages (CD11b + /Mac-1 +), CD45R0 T Memory cells, and perforin-positive cytotoxic cells (CD3 +) were analyzed and showed increased infiltrations.

**Fig. 1:**
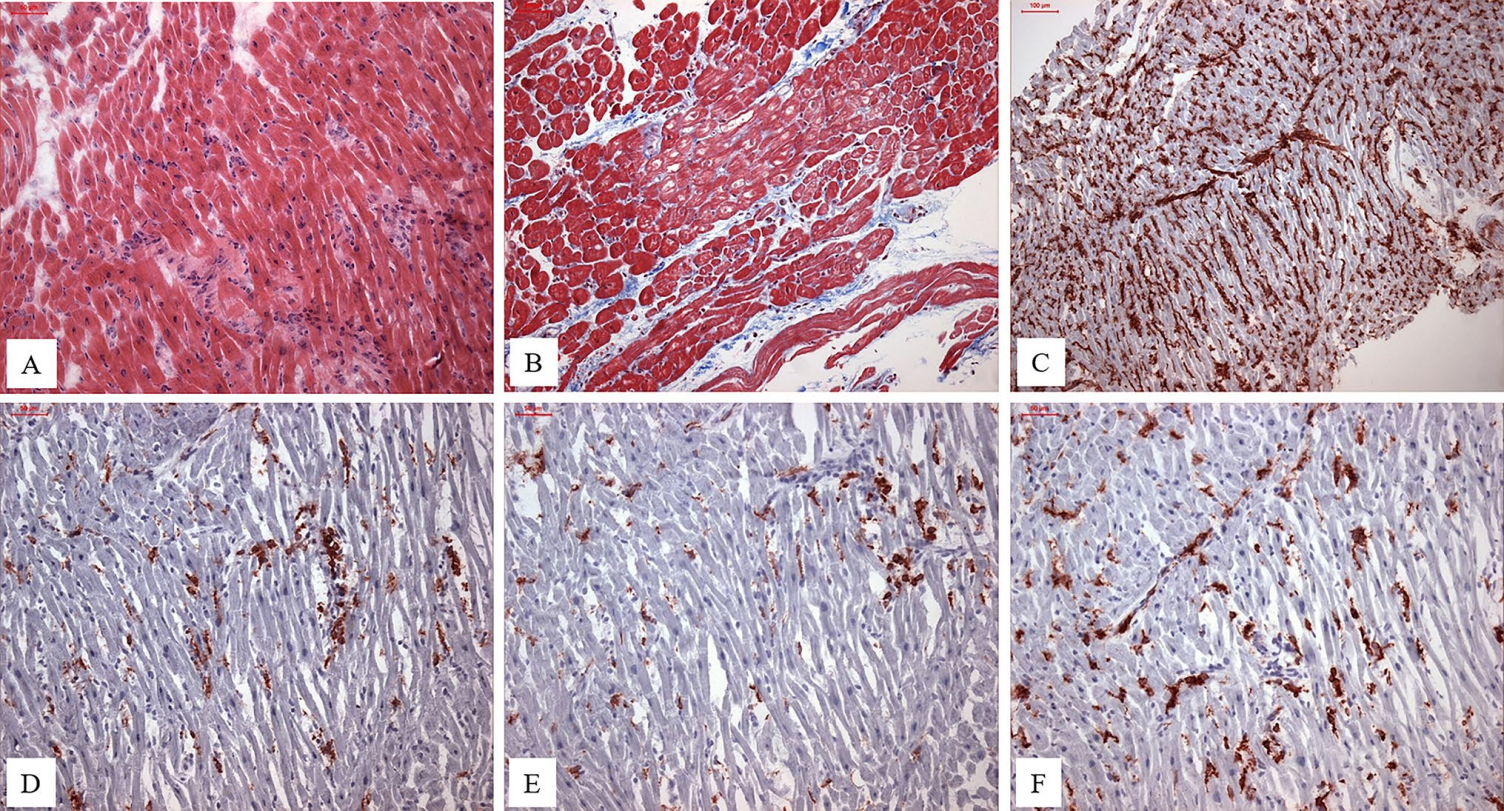
**A** Increased inflammatory cells as signs of active and chronic inflammation in a B19V-positive patient with transcriptional activity. Note the variation of myocyte diameters. H&E stain, bar 50 µm. **B** Increase of interstitial fibrosis (blue). PAS stain, bar 50 µm. **C** Immunohistochemical staining of increased Human Leukocyte Antigen—DR isotype—(HLA-DR) expression, bar 100 µm. **D** Immunohistochemical staining of pronounced increased diffuse infiltration of CD3-positive T lymphocytes, bar 50 µm. **E** Immunohistochemical staining of increased infiltration of CD11a-positive lymphocytes, bar 50 µm. **F** Immunohistochemical staining of pronounced increased infiltration of CD11b/MAC-1-positive macrophages, bar 50 µm

**Fig. 2:**
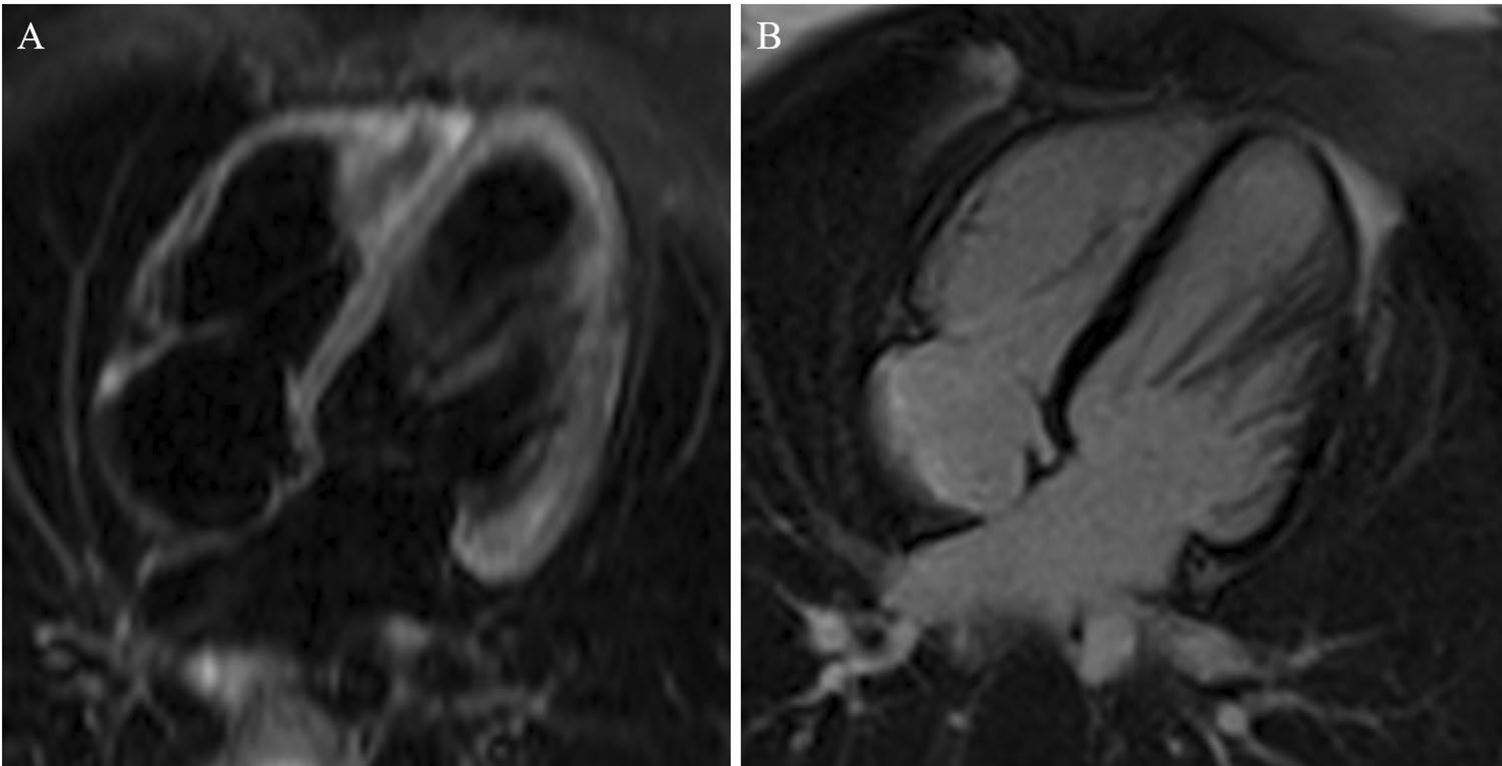
Cardiac MRI (4-chamber-view exemplarily) showing **A** no myocardial edema (T2-weighted edema images), and **B** no residual (T1-weighted) late gadolinium enhancement

Based on the clinical course, persistently reduced LV function and evidence of actively replicating B19V an antiviral therapy with tenofovir disoproxil (245 mg once daily) was initiated. Following step-wise reduction of pump-flow, the Impella pump was removed on day 8. Oral heart failure therapy was started including a beta-blocker, an angiotensin-converting enzyme inhibitor, and a mineralocorticoid-receptor antagonist. The patient remained in stable clinical condition. LV function steadily improved and was mildly reduced at discharge on day 17. On 3 month follow-up, LV function was completely normalized (Supplemental Video 2). There were no signs or symptoms of heart failure. No major adverse events occurred. NT-pro BNP level dropped to 167 ng/L. On 4 month follow-up, cardiac magnetic resonance imaging demonstrated the absence of late gadolinium enhancement and myocardial edema (Fig. 2). Due to complete recovery and physiological MRI findings no additional EMB has been performed.

We hypothesize that the patient developed severe heart failure due to immunosuppression caused by chemotherapy and subsequent activation of B19V replication that ultimately led to active myocarditis. Expression of both viral transcripts (NS1 and VP1/2) indicates B19V transcriptional activity. In contrast to latent infection (solely DNA genomes), expression of B19V viral RNA in the myocardium has been demonstrated to be of clinical significance [4, 5]. Treatment options of patients with acute lymphocytic myocarditis with LV dysfunction are poorly studied. Particularly, data on patients with acute B19V myocarditis are scarce. Treatment concepts include interferon-beta, immunoglobulins and antiviral therapy [6–8]. Van Linthout and colleagues previously reported 4 patients suffering from biopsy-proven chronic lymphocytic myocarditis and human B19V transcriptional activity who were treated with telbivudine (600 mg once daily for 6 months) that was initially approved to treat chronic hepatitis B virus infection [9]. After 6 months, endomyocardial biopsies showed decreased VP1/V2-mRNA levels and CD3 cells in all patients. This was associated with an improvement of LV function and New York Heart Association (NYHA) functional class. Consequently, the decision was made to initiate antiviral therapy. However, telbivudine is no longer available in Germany and an alternative was required. Telbivudine is a nucleoside analogue that inhibits viral DNA polymerase (reverse transcriptase). Additional pleiotropic immunomodulatory and anti-inflammatory effects have been shown [10]. Tenofovir also acts via nucleotide analogue reverse transcriptase inhibition and is approved for the treatment of HIV infection and chronic hepatitis B, but treatment of active B19V myocarditis with tenofovir has not yet been described. Because of similar pharmacological properties of telbivudine and tenofovir in the absence of therapeutic alternatives, we selected tenofovir disoproxil (245 mg once daily for 6 months) in combination with guideline-recommended heart failure therapy and temporary mechanical circulatory support [7, 11].

Acute viral myocarditis with cardiogenic shock early after chemotherapy is a rare and life-threatening entity. EMB can unveil the underlying pathology and guide diagnosis and specific therapy. Tenofovir disoproxil on top of guideline-recommended heart failure therapy demonstrated a safe and effective specific treatment option in this case of B19V-induced acute myocarditis.

## Supplementary Information

Below is the link to the electronic supplementary material.

Supplementary file1 Supplemental Video 1: Transthoracic echocardiogram at ICU admission (apical 4-chamber-view) showing a severely reduced systolic LV function and LV dilatation (AVI 876 KB)

Supplementary file2 Supplemental Video 2: Transthoracic echocardiogram at 3 months follow-up (apical 4-chamber-view) showing full recovery of systolic LV function. (AVI 1868 KB)

## Data Availability

All data and material are available and can be provided if requested.
